# The association between quality of life, intensity of counseling and health literacy amongst patients with nephrolithiasis

**DOI:** 10.1007/s00345-026-06336-x

**Published:** 2026-03-17

**Authors:** Wilson Sui, Lavanya Gupta, Al Sultan Azzawi, Maria C. Velasquez, Heiko Yang, Thomas Chi, Marshall L. Stoller

**Affiliations:** 1 Department of Urology, University of Michigan, 1500 E Medical Center Dr SPC 5330, Ann Arbor, MI 48109 USA; 2 College of Letters and Science, University of California Berkeley, Berkeley, CA USA; 3 Department of Urology, University of California San Francisco, San Francisco, CA USA

**Keywords:** Nephrolithiasis, Dietary prevention measures, Oxalate, Stone prevention, patient education

## Abstract

**Introduction:**

The American Urological Association (AUA) provides guidance on primary prevention for nephrolithiasis; however, patient compliance is often poor. The role of healthy literacy in the primary prevention of nephrolithiasis is not well understood. Understanding kidney stone formers’ perceptions and knowledge of primary prevention is critical to evaluating their health literacy. This study aimed to assess these perceptions and explore how they relate to counseling intensity and quality of life among patients with nephrolithiasis.

**Materials and Methods:**

A cross-sectional web-based survey was administered to a random sample of adult volunteers. Disease specific information was queried for stone formers and all participants were asked a series of questions based on the AUA Metabolic Stone Management Guidelines (2016) regarding primary prevention. Receipt of dietary counseling was categorized by comprehensiveness. Patient quality of life was assessed using the Wisconsin Stone Quality of Life questionnaire (WisQOL). Multivariable linear regression was used to identify predictors of stone-specific health literacy.

**Results:**

Of the 2482 participants, 429 (17%) reported prior stone history. Overall accuracy rates ranged from 10% to 98%. Stone patients with comprehensive counseling had the highest scores, driven by significantly better knowledge on oxalate-specific questions (*p* < 0.001). Multivariable linear regression showed receipt of comprehensive counseling (*p* = 0.032) was an independent predictor of higher primary prevention-specific health literacy.

**Conclusions:**

Overall, correct response rates were poor, especially surrounding dietary oxalates. However, kidney stone formers who received more comprehensive counseling demonstrated. greater disease-specific knowledge, serving as a proxy for improved health literacy.

**Supplementary Information:**

The online version contains supplementary material available at 10.1007/s00345-026-06336-x.

## Introduction

Nearly 1 in 11 people in the United States will develop nephrolithiasis and up to half will then develop a recurrent stone within 5 years [1,2]. The economic burden of the disease also continues to grow with stone treatment related expenses increasing 50% from 1994 to 2000 [3]. Prevention of subsequent stone events therefore has both clinical and financial implications. As the typical western diet – high in salt, animal protein and sweetened beverages – is associated with nephrolithiasis, primary prevention is based on dietary modifications [4–6]. Guideline recommendations include increasing fluid intake, maintaining a balanced calcium diet, minimizing dietary intake of animal protein and sodium and regulating oxalate intake [7–9].

Despite evidence that stone dietary prevention recommendations can be clinically efficacious, recurrent stone disease remains difficult to manage even with close follow up and motivated patients [10,11]. Barriers to primary prevention include provider-related factors such time constraints to counseling or failure to recommend appropriate treatment and patient-related factors such as difficulty with adherence or lack of access to healthy foods [12]. Health literacy – the capacity for patients to seek, understand and act on health information – is especially critical in management of chronic diseases where daily, consistent, long-lasting changes are necessary [13] Primary prevention of nephrolithiasis can involve multiple simultaneous changes to daily dietary habits which may be difficult for patients to understand and retain. In a study of community fair participants, fewer than half identified the influence 13 dietary items could have on stone risk [14] Amongst a cohort of stone formers, almost three-fourths did not know or did not believe dietary factors can influence stone formation [15].

In this context, this study sought to evaluate kidney stone patients’ understanding of dietary stone prevention strategies using a large, national survey collaborative. Secondary objectives included assessing the association between stone-specific quality of life measures and understanding of dietary prevention strategies.

## Materials and methods

### Recruitment and study materials

**Fig. 1:**
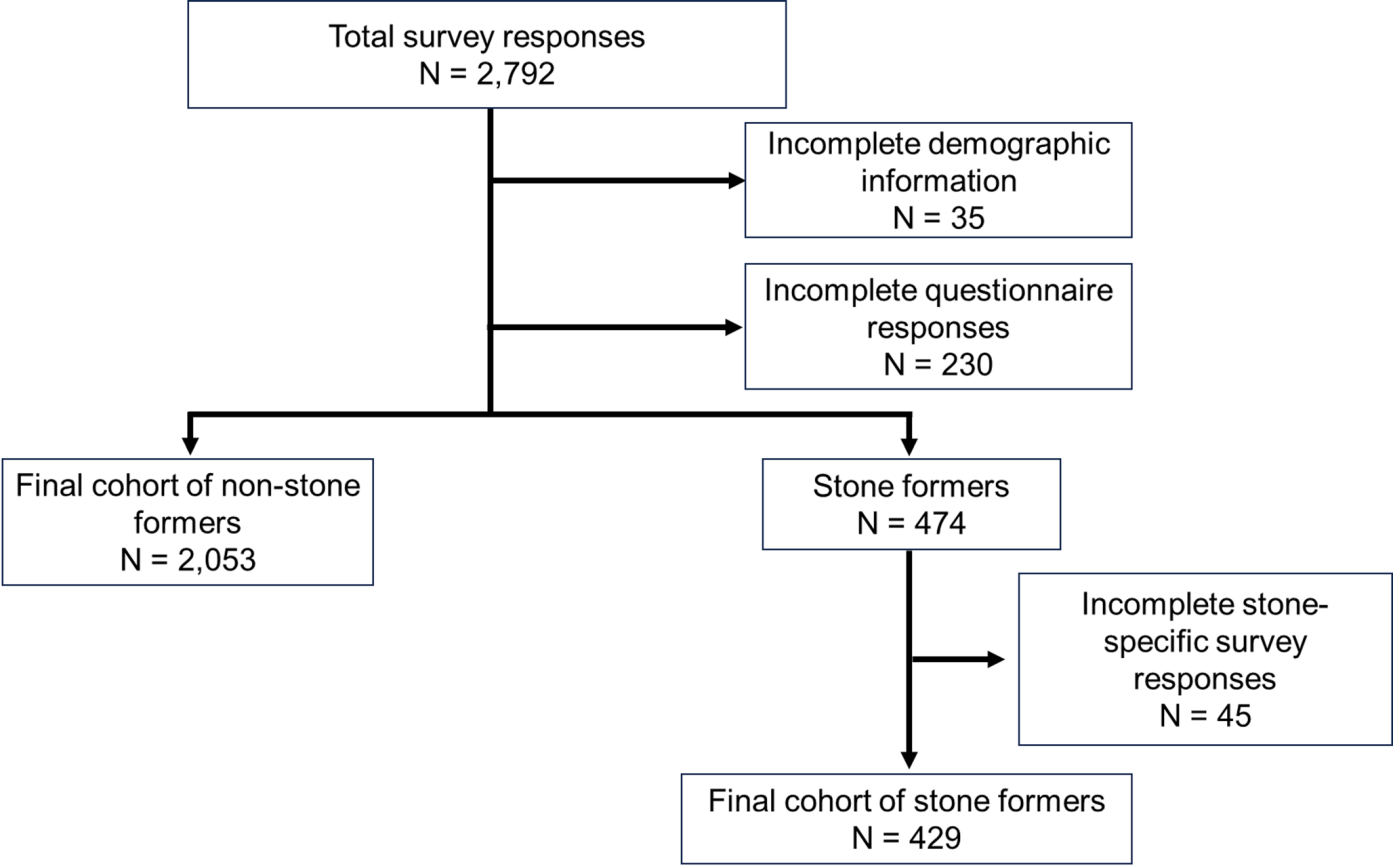
Cohort selection

Between February and September 2023, an anonymous survey was created in REDCap™ and then distributed via ResearchMatch™, a secure, national, online medical survey collaborative. Participants were incentivized with a chance to be 1 of 10 participants randomly selected to receive $50. Of the 17,221 possible participants contacted, 2,792 responded for a 16.2% response rate (Fig. 1). Institutional review board approval was obtained (IRB #23-38991) at the University of California, San Francisco.

A total of 65 questions were included (Supplementary materials). Assessments of patient demographic characteristics and past medical history were obtained from all participants. A screening question to confirm kidney stone history was included and these participants received additional questions regarding their stone-specific history including family history, prior surgeries, receipt of prior metabolic workup and receipt of dietary counseling. Nephrolithiasis-specific counseling was categorized by comprehensiveness – none (receipt of neither dietary counseling or a metabolic workup), partial (receipt of either dietary counseling or a metabolic workup) or comprehensive (receipt of both dietary counseling and a metabolic workup). The Wisconsin Stone Quality of Life (WisQOL) instrument was also used to measure disease-specific quality of life [16]. To evaluate participant understanding of dietary recommendations, participants were asked to classify various foods and beverages and their influence on stone growth. This questionnaire was adapted from a prior cohort study [14] and is based on AUA medical management of stones guidelines [7]. All questions were yes/no.

The primary outcome was to identify predictors of improved understanding of metabolic stone management. In order to understand baseline stone prevention health literacy knowledge in the community, non-stone formers were included in the initial survey. Each correct question was given one point with twelve being the maximum score. Patients who did not fill out the complete survey were removed from the analysis (Fig. 1). While not a validated general health literacy instrument, the dietary questionnaire served as a disease-specific proxy for assessing health literacy related to kidney stone prevention. Secondary outcomes included WisQOL scores for stone formers.

### Statistical analysis

Participants were categorized into non-stone formers and stone formers. Stone formers were then further categorized by comprehensiveness of stone-specific counseling (receipt of neither, either or both a metabolic workup and dietary counseling). Descriptive statistics were performed to compare demographic and disease-specific characteristics between the aforementioned categories. For continuous variables, analysis of variance was performed and for categorical variables, chi-square analysis was applied. Multivariable linear regression was performed to identify predictors of higher dietary stone prevention questionnaire scores amongst stone formers. The model included age, gender, race, education level, insurance status, history of diabetes, obesity, family history of kidney stones, recurrent stone formation, prior stone surgery, receipt of metabolic workup or dietary counseling and WisQOL scores. All statistics were performed using SPSS v27.

## Results

Overall, 2,053 non-stone formers and 429 stone formers responded to the survey (Fig. 1). Of the stone formers, 41% (177) reported no prior counseling, 37% (157) reported partial counseling and 21% (91) reported comprehensive counseling (Table 1). Amongst stone formers, participants who received comprehensive counseling were younger, more likely to be male and non-white, were more educated, more likely to be employed and have private insurance (all *p* < 0.05). These patients were also more likely to have a family history of stones, be a recurrent stone former and have undergone prior stone surgeries (*p* < 0.001).

**Table 1:**
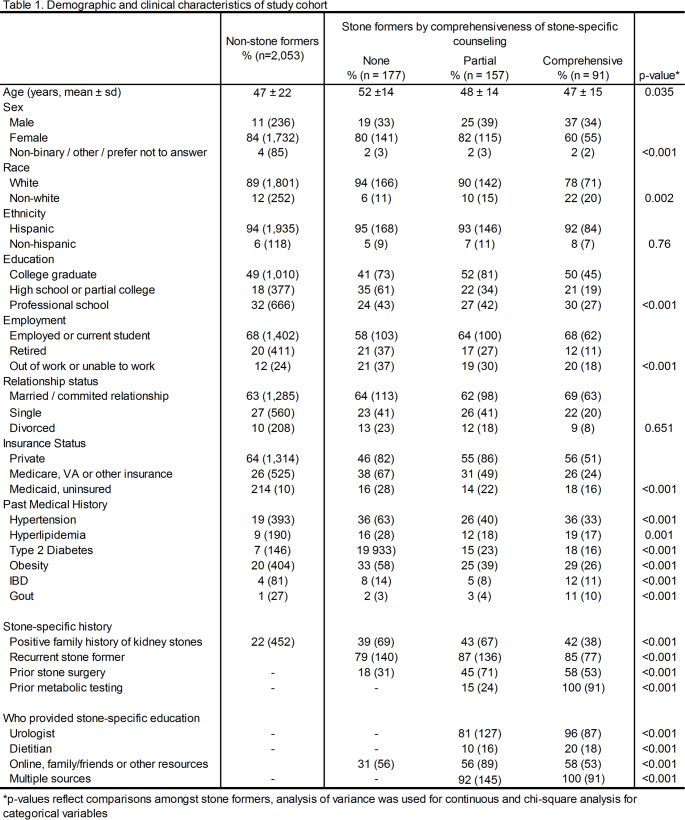

**Fig. 2:**
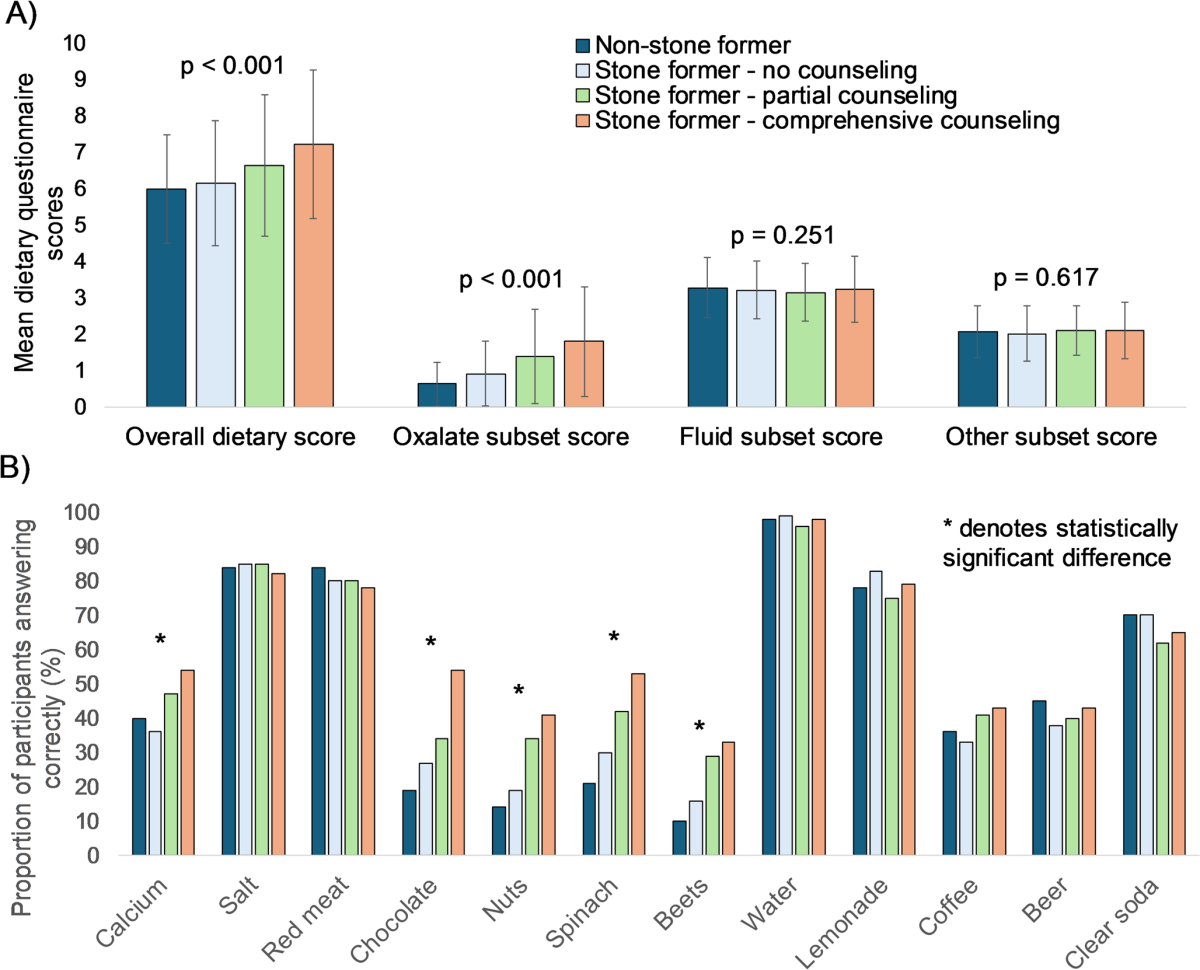
Comparison of **A** overall and subset scores and individual question performance **B** between non-stone formers and stone formers stratified by comprehensiveness of stone-specific counseling. Analysis of variance used to calculate individual *p*-values and *denotes statistically significant difference on chi-square analysis

Non-stone formers had the lowest accuracy (Fig. 2A), and amongst stone formers, those who received comprehensive counseling performed the best. The differences were driven by the subset of oxalate-related questions (*p* < 0.001) as there were no differences in mean scores on fluid-related questions (*p* = 0.251) or on the other remaining questions (*p* = 0.617). On assessment of individual questions, accuracy rates ranged from 10% to 98% (Fig. 2B). Over 60% answered questions regarding the role of water, salt, lemonade, red meat and clear soda correctly. However, in comparison, on the remaining seven questions accuracy ranged widely from 10% to 54%. Significant differences were identified on questions regarding calcium, chocolate, nuts, spinach and beets, with increasing comprehensiveness of counseling associated with better accuracy.

**Fig. 3:**
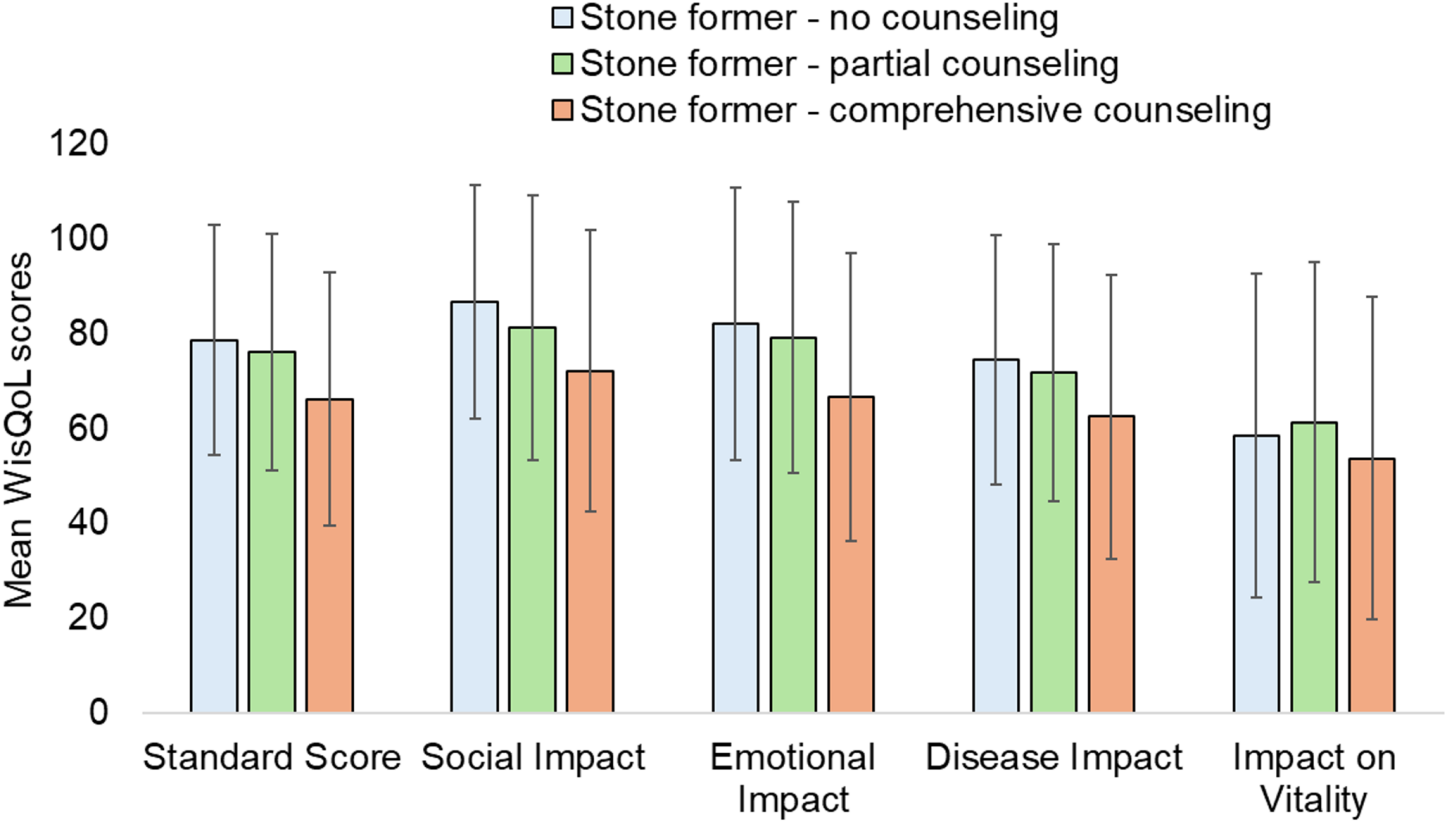
Mean WisQoL scores of stone formers stratified by comprehensiveness of stone-specific counseling

Stone formers who received neither counseling nor workup had the highest mean WisQOL scores compared to those who received either or both (Fig. 3). The same trend was observed in the social impact, emotional impact and disease impact domains while there were no differences in the impact on vitality measure.

On multivariable linear regression (Supplementary Table 1), predictors of improved understanding of dietary stone recommendations included younger age (β − 0.015, 95% CI − 0.03 to − 0.001), history of recurrent stones (β 0.548, 95% CI 0.052–1.044), having received both counseling or metabolic workup (β 0.559, 95% CI 0.049–1.07) and lower WisQOL scores (β − 0.01, 95% CI ± -0.018 to − 0.002).

## Discussion

Our study has two principal findings. One, while both stone formers and non-stone formers had similar understanding of the role that fluid, red meat and salt have on recurrent stone disease, stone formers demonstrated significantly better understanding of the role of dietary oxalate. Two, amongst stone formers, receipt of both dietary counseling and a metabolic workup was associated with the best understanding of dietary prevention strategies.

While stone formers demonstrated better understanding of dietary prevention strategies compared to non-stone formers, accuracy rates were poor overall. Amongst all participants, > 60% answered questions surrounding the role of water, salt, lemonade, red meat and clear soda correctly. On the remaining seven questions, however, accuracy ranged widely from 10% to 54%. Stone formers who received both counseling and underwent a metabolic workup, performed significantly better than non-stone formers and stone formers who received either forms of counseling or none on questions surrounding oxalate-containing foods. Given the specialized knowledge this represents, this suggests that dietary counseling is retained and is efficacious. However, it is notable that at least half of patients – including those who have received a metabolic workup and counseling – did not recall this information. These response rates were similar to those reported in a study of 753 participants recruited from a county fair, 35% of whom had a history of prior stone disease [14]. As in this prior study, our cohort was highly educated, with over 80% reporting at least a college degree. However, this finding highlights that kidney stone specific education encompasses specialized concepts not commonly encountered during general education, suggesting that baseline knowledge gaps persist regardless of formal educational attainment. Participants (regardless of prior stone history) identified the role of water and salt in stone formation most accurately. The role of oxalate-containing foods, however, was the most challenging, with less than 50% accuracy rates across all items. These findings suggest that oxalate-specific education could be a high yield target for future patient education interventions though concerns remain regarding the efficacy of such dietary interventions in decreasing stone recurrence.

Both stone-specific quality of life and intensity of counseling were predictors of disease-specific health literacy. In our multivariable model, receipt of both a workup and counseling were associated with increased stone-specific health literacy (Supplementary Table 1). Stone formers who received more counseling also demonstrated worse quality of life, likely because these patients had more severe underlying stone disease as demonstrated by a higher proportion of patients who received more counseling also reporting prior surgery or recurrent stones. To our knowledge, this is the first report of this association amongst stone patients though this is not surprising as health literacy has been shown to be positively correlated with quality of life amongst cancer patients [17] and other chronic diseases.^18^,^19^ In addition, low health literacy has been associated with poor health outcomes and up to half of patients are at risk of limited health literacy [20, 21]. Health literacy regarding stone disease may be limited as in a cross-sectional study of 1,018 urologic patients, almost 75% did not believe or did not know diet influenced stone disease risk [15]. Comprehensive counseling and workup can improve patient understanding of their disease and encouragingly, over 70% of participants reported they would be ready to make dietary modifications to decrease their risk of stone formation [15]. However, long-term adherence, patient access to healthy foods, and other social determinants of health remain difficult barriers to overcome [12].

This study has several important limitations to consider. First, while our study participants were drawn from a large, nationwide, diverse population, it was still skewed towards white adults with at least a college education, limiting its generalizability. Patients with more education are less likely to present with severe stone disease 22, therefore, our cohort may be skewed more towards individuals with less advanced stone disease for whom a metabolic workup was not indicated. However, all patients with stone disease should receive at least stone dietary counseling and yet 42% of our respondents did not recall receiving either counseling or a workup. Second, our questionnaire was not validated so there is a risk of measurement bias in our sample. The dietary questionnaire served as a disease-specific proxy for assessing health literacy related to kidney stone prevention. In addition, as a survey-based study, there is a chance of recall bias or response error. However, our survey is based on one used in a community cohort [14] and yielded similar accuracy rates among similar domains. Third, the recommendations tested were not as applicable to non-calcium-based stones (i.e. uric acid or struvite), however, calcium-based stones are by far the most common amongst stone formers [23] We also did not have patients’ stone composition though self-reported stone composition would be prone to recall bias. Future directions could incorporate these data in order to better understand dietary education gaps. Fourth, while we found the largest differences in accuracy amongst oxalate-related questions, many standard stone patients would not be expected to have received this targeted counseling as oxalate is not a component of all stones and hyperoxaluria requires a 24-hour urine test to identify. Fifth, our study did not use a standardized health literacy tool, and instead used a knowledge-based questionnaire as a proxy to assess disease-specific understanding. Finally, our questionnaire may be over-simplified especially with only a binary outcome where there are many nuances to stone dietary management. While this may limit our ability to capture partial understanding, prior research suggests that a good-bad dichotomy may be the most effective for dietary counseling [24].

Nonetheless, this study is the largest reported in the literature with over 2400 respondents sampled from across the nation and incorporated assessment of disease-specific quality of life using a validated survey [16]. The overall low accuracy rate for both non-stone formers and stone formers on oxalate-specific questions in particular suggests this may be a high yield target for future intervention. While adherence rates to dietary therapy are commonly reported below 50% [25, 26], strategies for improving patient adherence have included incorporating Registered Dietitian Nutritionists into practice [27] and group appointments for stone patients [28]. The rise of smart or digital health technology [29,30] may improve patient adherence with smart containers to monitor fluid intake, wearable technology with sensor capability or applications to send automated electronic reminders. Ultimately, enabling stone patients to better understand their own disease should lead to improved participation in and adherence to primary prevention thereby leading to decreased recurrent stone events and improve quality of life.

## Conclusions

Overall understanding of primary prevention strategies is especially poor surrounding dietary oxalates. However, kidney stone formers who received more comprehensive counseling demonstrated greater disease-specific knowledge, serving as a proxy for improved health literacy. Future interventions centered around understanding sources of dietary oxalate could have the highest impact on primary prevention.

## Supplementary Information

Below is the link to the electronic supplementary material.

Supplementary Material 1

Supplementary Material 2

Supplementary Material 3

## Data Availability

Data is provided within the manuscript or supplementary information files.

## Abbreviations

AUA: American Urologic Association
EAU: European Association of Urology
WisQOL: Wisconsin Stone Quality of Life questionnaire

## References

[1] Scales CD, Smith AC, Hanley JM, Saigal CS (2012) Prevalence of Kidney Stones in the United States. Eur Urol 62(1):160–165. 10.1016/j.eururo.2012.03.052 22498635 10.1016/j.eururo.2012.03.052 PMC3362665

[2] Rule AD, Lieske JC, Li X, Melton LJ III, Krambeck AE, Bergstralh EJ (2014) The ROKS nomogram for predicting a second symptomatic stone episode. J Am Soc Nephrol 25(12):2878–2886 25104803 10.1681/ASN.2013091011 PMC4243346

[3] Pearle MS, Calhoun EA, Curhan GC (2005) Urologic diseases of America P. Urologic diseases in America project: urolithiasis. J Urol Mar 173(3):848–857. 10.1097/01.ju.0000152082.14384.d7 10.1097/01.ju.0000152082.14384.d7 15711292

[4] Fink HA, Akornor JW, Garimella PS et al (2009) Diet, fluid, or supplements for secondary prevention of nephrolithiasis: a systematic review and meta-analysis of randomized trials. Eur Urol 56(1):72–80 19321253 10.1016/j.eururo.2009.03.031 PMC2925677

[5] Han H, Segal AM, Seifter JL, Dwyer JT (2015) Nutritional management of kidney stones (nephrolithiasis). Clin Nutr Res 4(3):137–152 26251832 10.7762/cnr.2015.4.3.137 PMC4525130

[6] Sorensen MD, Kahn AJ, Reiner AP et al (2012) Impact of nutritional factors on incident kidney stone formation: a report from the WHI OS. J Urol May 187(5):1645–1649. 10.1016/j.juro.2011.12.077 10.1016/j.juro.2011.12.077 PMC4165387 22425103

[7] Pearle MS, Goldfarb DS, Assimos DG et al (2014) Medical management of kidney stones: AUA Guideline. J Urol Aug 192(2):316–324. 10.1016/j.juro.2014.05.006 10.1016/j.juro.2014.05.006 24857648

[8] Skolarikos A, Straub M, Knoll T et al (2015) Metabolic evaluation and recurrence prevention for urinary stone patients: EAU guidelines. Eur Urol Apr 67(4):750–763. 10.1016/j.eururo.2014.10.029 10.1016/j.eururo.2014.10.029 25454613

[9] Ferraro PM, Bargagli M, Trinchieri A, Gambaro G (2020) Risk of kidney stones: influence of dietary factors, dietary patterns, and vegetarian–vegan diets. Nutrients 12(3):779 32183500 10.3390/nu12030779 PMC7146511

[10] Hosking DH, Erickson SB, Van den Berg CJ, Wilson DM, Smith LH (1983) The stone clinic effect in patients with idiopathic calcium urolithiasis. J Urol Dec 130(6):1115–1118. 10.1016/s0022-5347(17)51711-5 10.1016/s0022-5347(17)51711-5 6644890

[11] Parks JH, Coe FL (2009) Evidence for durable kidney stone prevention over several decades. BJU Int May 103(9):1238–1246. 10.1111/j.1464-410X.2008.08170.x 10.1111/j.1464-410X.2008.08170.x PMC2768493 19021617

[12] Scotland KB, Armas-Phan M, Dominique G, Bayne D (2022) Social determinants of kidney stone disease: the impact of race, income and access on urolithiasis treatment and outcomes. Urology 163:190–195 34506806 10.1016/j.urology.2021.08.037 PMC9817034

[13] Nutbeam D (2008) The evolving concept of health literacy. Soc Sci Med 67(12):2072–2078 18952344 10.1016/j.socscimed.2008.09.050

[14] Marsh BM, Sathianathen N, Tejpaul R, Albersheim-Carter J, Bearrick E, Borofsky MS (2019) Public perceptions on the influence of diet and kidney stone formation. J Endourol May 33(5):423–429. 10.1089/end.2019.0010 10.1089/end.2019.0010 30880445

[15] Fakhoury MQ, Gordon B, Shorter B et al (2019) Perceptions of dietary factors promoting and preventing nephrolithiasis: a cross-sectional survey. World J Urol Aug 37(8):1723–1731. 10.1007/s00345-018-2562-6 10.1007/s00345-018-2562-6 30554273

[16] Penniston KL, Nakada SY (2013) Development of an instrument to assess the health related quality of life of kidney stone formers. J Urol 189(3):921–930 23017521 10.1016/j.juro.2012.08.247

[17] Halverson JL, Martinez-Donate AP, Palta M et al (2015) Health literacy and health-related quality of life among a population-based sample of cancer patients. J health communication 20(11):1320–1329 26161549 10.1080/10810730.2015.1018638 PMC4751057

[18] Sayah FA, Qiu W, Johnson JA (2016) Health literacy and health-related quality of life in adults with Type 2 diabetes: a longitudinal study. Qual Life Res 25:1487–1494 26603739 10.1007/s11136-015-1184-3

[19] Wolf MS, Davis TC, Osborn CY, Skripkauskas S, Bennett CL, Makoul G (2007) Literacy, self-efficacy, and HIV medication adherence. Patient Educ Couns 65(2):253–260 17118617 10.1016/j.pec.2006.08.006

[20] DeWalt DA, Pignone MP (2005) Reading is fundamental: the relationship between literacy and health. Arch Intern Med 165(17):1943–1944 16186462 10.1001/archinte.165.17.1943

[21] Adams RJ, Appleton SL, Hill CL, Dodd M, Findlay C, Wilson DH (2009) Risks associated with low functional health literacy in an Australian population. Med J Aust 191(10):530–534 19912083 10.5694/j.1326-5377.2009.tb03304.x

[22] Bayne DB, Usawachintachit M, Armas-Phan M et al (2019) Influence of socioeconomic factors on stone burden at presentation to tertiary referral center: data from the registry for stones of the kidney and ureter. Urology Sep 131:57–63. 10.1016/j.urology.2019.05.009 10.1016/j.urology.2019.05.009 PMC6711808 31132427

[23] Herring LC (1962) Observations on the analysis of ten thousand urinary calculi. J Urol Oct 88:545–562. 10.1016/S0022-5347(17)64842-0 10.1016/S0022-5347(17)64842-0 13954078

[24] Oakes ME (2005) Stereotypical thinking about foods and perceived capacity to promote weight gain. Appetite Jun 44(3):317–324. 10.1016/j.appet.2005.03.010 10.1016/j.appet.2005.03.010 15896876

[25] Khambati A, Matulewicz RS, Perry KT, Nadler RB (2017) Factors associated with compliance to increased fluid intake and urine volume following dietary counseling in first-time kidney stone patients. J Endourol 31(6):605–610 28318298 10.1089/end.2016.0836

[26] van Drongelen J, Kiemeney LA, Debruyne FM, de la Rosette JJ (1998) Impact of urometabolic evaluation on prevention of urolithiasis: a retrospective study. Urology 52(3):384–391 9730448 10.1016/s0090-4295(98)00201-5

[27] Jhagroo RA, Nakada SY, Penniston KL (2013) Shared medical appointments for patients with kidney stones new to medical management decrease appointment wait time and increase patient knowledge. J Urol 190(5):1778–1784 23707453 10.1016/j.juro.2013.05.037

[28] Beto JA, Ramirez WE, Bansal VK (2014) Medical nutrition therapy in adults with chronic kidney disease: integrating evidence and consensus into practice for the generalist registered dietitian nutritionist. J Acad Nutr Dietetics 114(7):1077–1087 10.1016/j.jand.2013.12.009 24582998

[29] Wright HC, Alshara L, DiGennaro H et al (2022) The impact of smart technology on adherence rates and fluid management in the prevention of kidney stones. Urolithiasis Feb 50(1):29–36. 10.1007/s00240-021-01270-6 10.1007/s00240-021-01270-6 34115205

[30] Streeper NM, Dubnansky A, Sanders AB, Lehman K, Thomaz E, Conroy DE (2019) Improving fluid intake behavior among patients with kidney stones: understanding patients’ experiences and acceptability of digital health technology. Urology Nov 133:57–66. 10.1016/j.urology.2019.05.056 10.1016/j.urology.2019.05.056 31374289

