# Supplementary material for: The association between quality of life, intensity of counseling and health literacy amongst patients with nephrolithiasis
Source: World J Urol. 2026 Mar 17;44(1):248. doi: 10.1007/s00345-026-06336-x (PMC12995978; doi:10.1007/s00345-026-06336-x)
Supplement: Supplementary file 1 — Supplementary Material 1 [file 345_2026_6336_MOESM1_ESM.pdf]

This section of the questionnaire is designed to understand the quality of life of patients with a history of kidney stones. The questions below ask about how problems with kidney stones have affected you during the past month. Some questions may look very similar or have similar wording, but each one is different. Please answer the questions as honestly as possible. Although you may have a number of physical or medical problems, please do your best to think only about your problems related to kidney stones.

In the last four weeks how true for you are the following statements?

		Very true	Mostly true	Somewhat true	A little true	Not at all true
48)	My energy level during the day is less than usual	○	○	○	○	○
49)	I feel very tired or fatigued	○	○	○	○	○
50)	My activity is limited	○	○	○	○	○

Because of kidney stones, how true have any of these problems been for you within the last 4 weeks?

		Very true	Mostly true	Somewhat true	A little true	Not at all true
51)	Trouble getting to sleep or with waking up while trying to sleep	○	○	○	○	○
52)	Needing to get up frequently while sleeping to urinate	○	○	○	○	○
53)	Poor quality sleep or not feeling rested after sleeping	○	○	○	○	○
54)	Difficulty returning to sleep	○	○	○	○	○

Because of kidney stones, how true for you over the last 4 weeks are the following?

		Very true	Mostly true	Somewhat true	A little true	Not at all true
55)	I don't feel the usual freedom to travel or to attend or to participate in social events	○	○	○	○	○
56)	I force myself to go to work or school, to exercise, or to fulfill other responsibilities	○	○	○	○	○
57)	I have missed work or family time, or lost leisure or recreation time	○	○	○	○	○
58)	I make frequent changes or adjustments to my daily schedule	○	○	○	○	○
59)	I have less ability than usual to focus on my work, family, or other commitments or interests	○	○	○	○	○

**How often have you experienced or felt the following in the last 4 weeks because of kidney stones?**	Always or almost always	Very often	Somewhat often	Hardly at all	Not at all, never
60) Problems or difficulty sticking to the diet recommendations	○	○	○	○	○
61) Problems tolerating or taking prescription medications as directed	○	○	○	○	○
62) Concern about my general health	○	○	○	○	○

**Below are some physical symptoms that might be related to kidney stones. In the last four weeks, how often have you felt these symptoms?**	Always or almost always	Very often	Somewhat often	Hardly at all	Not at all, never
63) Nausea, stomach upset, or cramps	○	○	○	○	○
64) Physical pain	○	○	○	○	○
65) Urinary frequency (feeling like you have to go more than usual)	○	○	○	○	○
66) Urinary urgency (sudden or unstoppable urge to urinate)	○	○	○	○	○

**Because of kidney stones, in the last four weeks, how true are the following for you?**	Very true	Mostly true	Somewhat true	A little true	Not at all true
67) I have less interest in sex or less sexual contact than usual	○	○	○	○	○
68) I need to make special arrangements when traveling	○	○	○	○	○
69) I have less interest than usual in socializing/being around others	○	○	○	○	○

**In the last four weeks, because of your kidney stones, how much have you felt the following?**	Very much	Quite a lot	Somewhat	A little bit	Not at all, never
70) Frustrated with my situation	○	○	○	○	○
71) Worried about what is wrong now	○	○	○	○	○
72) Anxious or nervous about what might go wrong in the future	○	○	○	○	○
73) Annoyed at the nuisances and inconveniences of my situation	○	○	○	○	○
74) Reduced ability, compared to usual, to cope with everyday issues or responsibilities	○	○	○	○	○

75)

More irritable than usual	○	○	○	○	○

76) Standing Total

____

77) Social Impact

____

78) Emotional Impact

____

79) Disease Impact

____

80) Impact on Vitality

____
