# Supplementary material for: The association between quality of life, intensity of counseling and health literacy amongst patients with nephrolithiasis
Source: World J Urol. 2026 Mar 17;44(1):248. doi: 10.1007/s00345-026-06336-x (PMC12995978; doi:10.1007/s00345-026-06336-x)
Supplement: Supplementary file 2 — Supplementary Material 2 [file 345_2026_6336_MOESM2_ESM.pdf]

# Demographics And Kidney Stone Medical History

This survey is a UCSF kidney stone research project studying the public perceptions of kidney stone disease. If you have no history of kidney stones, we would still like to know about your perceptions of the disease.

We would like to know your understanding of dietary preventative strategies for kidney stones as well as experiences with kidney stone disease (if you have had them).

Please complete this 15-minute survey (your responses will be kept anonymous). Thank you for your participation!

| | |
|---|---|
| Have you previously been told you have kidney stones by your doctor OR passed a kidney stone OR had surgery for kidney stones? | ◯ Yes<br>◯ No |
| Please continue the survey. Your responses are still valuable to understand perceptions on Kidney Stones. | |
| Age | __________________________ |
| Ethnicity: Are you of Hispanic, Latino/a, or Spanish origin? | ◯ Yes<br>◯ No<br>◯ Prefer not to answer |
| Race: What is your race? (Select all that apply) | ☐ American Indian or Alaska Native<br>☐ Asian<br>☐ Black or African American<br>☐ Native Hawaiian or Pacific Islander<br>☐ White<br>☐ Other: (Please specify)<br>☐ Prefer not to answer |
| If you selected "other" for the previous question, specify your race: | __________________________ |
| What gender do you identify as? | ◯ Male<br>◯ Female<br>◯ Gender non-conforming/Non-binary<br>◯ Gender fluid<br>◯ Other<br>◯ Prefer not to answer |
| Please specify if you selected "other" for the previous question | __________________________ |
| Please select your relationship status: | ◯ Single<br>◯ Married<br>◯ In a Relationship, living with partner<br>◯ In a Relationship, not living with partner Engaged<br>◯ Divorced/Separated |
| Education Level | ◯ Did not graduate high school<br>◯ High School Graduate<br>◯ Some College<br>◯ College Graduate<br>◯ Professional School Graduate |

| | |
|---|---|
| I have been diagnosed with the following conditions (select all that apply): | ☐ Hypertension<br>☐ Hyperlipidemia<br>☐ Diabetes<br>☐ Obesity<br>☐ COPD<br>☐ Heart Attack<br>☐ Stroke<br>☐ Inflammatory Bowel Disease (i.e. Crohn's Disease, Gout)<br>☐ Gout<br>☐ Cystinuria |
| Please enter your employment status | ◯ Employed for wages<br>◯ Self-employed<br>◯ Out of work and looking for work<br>◯ Out of work but not currently looking for work<br>◯ A student<br>◯ Military<br>◯ Retired<br>◯ Unable to work |
| Please select your CURRENT type of health insurance | ◯ Uninsured<br>◯ Private Insurance/HMO<br>◯ Medicare<br>◯ Medicaid<br>◯ VA<br>◯ Other |
| Have you passed any kidney stones naturally (on your own, without surgery)? | ◯ Yes<br>◯ No |
| How many kidney stones have you passed naturally (on your own, without surgery) IN THE LAST YEAR? | ______________________ |
| How many kidney stones have you passed in your lifetime? | ______________________ |
| Have you had any surgeries for kidney stone treatment? | ◯ Yes<br>◯ No |
| How many surgeries have you had for kidney stone treatment IN THE LAST YEAR (extraction, removal, or fragmenting) ? | ______________________ |
| How many surgeries have you had for kidney stone treatment in your lifetime (laser or shockwave treatment)? | ______________________ |

Medical history questions: Please answer according to your medical history. All of your responses are confidential.

| | |
|---|---|
| Have you learned about Kidney Stone prevention from urologists, dieticians, online resources or friends/family to prevent kidney stones? | ◯ Yes<br>◯ No |

| | |
|---|---|
| Who or where did you learn about Kidney Stone prevention from? (Select all that apply) | ☐ Urologists<br>☐ Dietician<br>☐ Online<br>☐ Friends/Family<br>☐ Others |
| Do you have a family history of kidney stones? (First degree relative) | ◯ Yes<br>◯ No<br>◯ Not Sure |
| Have you ever undergone testing to identify risk factors for kidney stone recurrence? (blood or urine test) | ◯ Yes<br>◯ No<br>◯ Not Sure |
| Compared to other people your age, would you say you are more physically active, less active, or about as active? | ◯ More active<br>◯ Less active<br>◯ Similar |

# Kidney Stone Perceptions

These questions will test your general knowledge regarding kidney stone prevention.

| | | |
|---|---|---|
| 1 | Does a moderate amount of calcium decrease the risk of kidney stones? | ◯ Yes<br>◯ No |
| 2 | Does increased salt decrease stone recurrence? | ◯ Yes<br>◯ No |
| 3 | Is red meat worse than white meat? | ◯ Yes<br>◯ No |
| 4 | Does chocolate promote stone growth? | ◯ Yes<br>◯ No |
| 5 | Do nuts promote stone growth? | ◯ Yes<br>◯ No |
| 6 | Does spinach promote stone growth? | ◯ Yes<br>◯ No |
| 7 | Do beets promote stone growth? | ◯ Yes<br>◯ No |
| 8 | Does water promote stone growth? | ◯ Yes<br>◯ No |
| 9 | Does lemonade promote stone growth? | ◯ Yes<br>◯ No |
| 10 | Does coffee promote stone growth? | ◯ Yes<br>◯ No |
| 11 | Does beer promote stone growth? | ◯ Yes<br>◯ No |
| 12 | Does clear soda promote stone growth? | ◯ Yes<br>◯ No |
| | My chance of having another kidney stone within 10 years is: | ◯ 0%<br>◯ 20%<br>◯ 40%<br>◯ 60%<br>◯ 100% |
| | My chance of having a kidney stone within 10 years is: | ◯ 0%<br>◯ 20%<br>◯ 40%<br>◯ 60%<br>◯ 100% |
