# Supplementary material for: The association between quality of life, intensity of counseling and health literacy amongst patients with nephrolithiasis
Source: World J Urol. 2026 Mar 17;44(1):248. doi: 10.1007/s00345-026-06336-x (PMC12995978; doi:10.1007/s00345-026-06336-x)
Supplement: Supplementary file 3 [file 345_2026_6336_MOESM3_ESM.docx]

Supplementary Table 1. Multivariable linear regression to identify predictors of patient understanding of dietary stone prevention recommendations

	β (95% CI)	p-value
Age (years, mean ± sd)	-0.015 (-0.03 - -0.001)	0.032
Sex		
Female	ref	
Non-female	0.366 (-0.257 - 0.989)	0.249
Race		
White	ref	
Non-white	-0.4 (-0.836 - 0.036)	0.072
Education		
College graduate	ref	
High school or partial college	-0.01 (-0.459 - 0.439)	0.966
Professional school	0.339 (-0.098 - 0.776)	0.128
Insurance Status		
Private	ref	
Medicare, VA or other insurance	-0.169 (-0.603 - 0.266)	0.446
Medicaid, uninsured	-0.007 (-0.557 - 0.542)	0.979
Type 2 Diabetes	-0.093 (-0.597 - 0.411)	0.717
Obesity	-0.035 (-0.446 - 0.376)	0.867
Family history of kidney stones	0.168 (-0.204 - 0.539)	0.376
Recurrent stone former	0.548 (0.052 - 1.044)	0.030
Prior stone surgery	0.06 (-0.349 - 0.468)	0.774
Stone-specific counseling		
None	ref	
Partial	0.124 (-0.297 - 0.545)	0.562
Comprehensive	0.559 (0.049 - 1.07)	0.032
WisQOL standard score	-0.01 (-0.018 - -0.002)	0.017
